# Multifactorial Origin of Neurodevelopmental Disorders: Approaches to Understanding Complex Etiologies

**DOI:** 10.3390/toxics3010089

**Published:** 2015-03-23

**Authors:** Alessia De Felice, Laura Ricceri, Aldina Venerosi, Flavia Chiarotti, Gemma Calamandrei

**Affiliations:** Unit of Neurotoxicology and Neuroendocrinology, Department of Cell Biology and Neurosciences, Istituto Superiore di Sanità, Viale Regina Elena 299, I-00161 Roma, Italy; E-Mails: alessiadefelice5@gmail.com (A.D.F.); laura.ricceri@iss.it (L.R.); aldina.venerosi@iss.it (A.V.); flavia.chiarotti@iss.it (F.C.)

**Keywords:** developmental neurotoxicity, autism spectrum disorders, attention deficit hyperactivity disorder, methylmercury, organophosphate pesticides, single nucleotide polymorphisms, maternal stress, exposome, biomarkers, rodents

## Abstract

A significant body of evidence supports the multifactorial etiology of neurodevelopmental disorders (NDDs) affecting children. The present review focuses on early exposure to environmental chemicals as a risk factor for neurodevelopment, and presents the major lines of evidence derived from epidemiological studies, underlying key uncertainties and research needs in this field. We introduce the exposome concept that, encompassing the totality of human environmental exposures to multiple risk factors, aims at explaining individual vulnerability and resilience to early chemical exposure. In this framework, we synthetically review the role of variable gene backgrounds, the involvement of epigenetic mechanisms as well as the function played by potential effect modifiers such as socioeconomic status. We describe laboratory rodent studies where the neurodevelopmental effects of environmental chemicals are assessed in the presence of either a “vulnerable” gene background or adverse pregnancy conditions (*i.e.*, maternal stress). Finally, we discuss the need for more descriptive and “lifelike” experimental models of NDDs, to identify candidate biomarkers and pinpoint susceptible groups or life stages to be translated to large prospective studies within the exposome framework.

## 1. Introduction

The brain has a protracted period of susceptibility to environmental inputs, which extends well beyond organogenesis up to the second decade of life [1]. Brain development requires a series of sequential and interacting steps, each controlled by intrinsic developmental processes that are modulated by external influences. The primary event in the formation of the central nervous system (CNS) is the appearance of the neural plate from the ectoderm, during the second and the third weeks of gestational age [2], followed by rapid cell proliferation as the plate folds to form the neural tube. Cells formed during this period of intense proliferation are known as neuroblasts and extend in the ventricular zone, where they continue to proliferate and subsequently migrate from this region toward their final destination with the help of the radial glial cells [3], depending on the presence of different molecular gradients in their location [4,5]. In the human fetus, cell migration is nearly complete in the neocortex and in most of the brain by the sixth month of gestation [6].

During the remainder of intrauterine development, neurons differentiate and connect to each other, and these two processes (differentiation and synapse formation) continue for several years after birth. Synapse formation is the final step in the establishment of CNS circuitry: substantially more connections are formed than those that will be eventually retained as many of them will be gradually discarded. The developing brain is characterized by many waves of apoptosis that control the processes of creating, strengthening, and discarding connections among the neurons; these processes continue into early postnatal years in response to the young child’s experiences. The remodeling of synaptic connections to establish functional properties of cortical circuits requires also the glial component of white matter that supports the neural migration, the regulation of extracellular environment and the synaptic connections [7]. The process of synapse elimination is a normal part of development [7,8] and both endogenous (neurotrophic factors, synthesis and release of neurotransmitters, hormones) and environmental factors (*i.e.*, sensorial inputs) concur to influence the fine matchup between pre- and postsynaptic neurons.

Within the constraints posed by the genome, the brain receives information from the environment and uses this information to shape and refine synaptic connections. In such a way, experience adjusts the underlying brain circuitry founded on the distinctive environment in which each individual lives and grows up. This has been clearly demonstrated by pioneering experiments showing the fundamental role of sensory input during critical periods of development for appropriate formation of connections in the visual cortex [9]. Furthermore, the evidence that growing up in enriched environments produces experience-induced alterations in brain morphology in rats (reviewed in [10]) demonstrated that also in the presence of subtle changes in the environment or habits, experience-dependent neural activity plays critical roles in brain development.

Vulnerability to adverse environmental factors represents the drawback of brain plasticity. The dynamic interplay between genes and environment, which forms the basis of typical neurobehavioral maturation, is being also called upon to explain the etiology of complex neurodevelopmental disorders (NDDs) that are characterized by abnormal brain morphology and/or functional activity, even those with a strong genetic component. Diverse environmental stressors—chemical pollutants, drugs, nutritional factors, maternal infection, stress, deprivation—may interfere with typical brain developmental trajectories, eventually increasing the risk of either subclinical neuropsychological alterations or manifest clinical conditions such as learning disabilities, autism spectrum disorders (ASD) and attention deficit/hyperactivity disorder (ADHD).

The present review focuses on early exposure to environmental chemicals. First, we summarize the major lines of evidence derived from epidemiological studies that suggest association between exposure to chemicals and altered neurodevelopment in children, underlying key uncertainties and research needs in the field of environmental origin of major human NDDs. We then introduce the exposome approach, which investigates the relationship between all the exposures of an individual in a lifetime and her/his health outcome, to explain the variability in individual vulnerability and resilience to early chemical exposure. In this framework, we synthetically review the role of variable gene backgrounds, the involvement of epigenetic mechanisms as well as the function played by potential effect modifiers such as socioeconomic status (SES). In the second part of this review, we describe laboratory rodent studies where the neurodevelopmental effects of environmental chemicals are assessed in the presence of either a “vulnerable” gene background or adverse pregnancy conditions (*i.e.*, infective state, maternal stress). Finally, we discuss the need for more descriptive and “lifelike” experimental models of NDDs, in the framework of the new challenges posed by the exposome concept to environmental health research.

## 2. Environmental Chemicals and Neurobehavioral Toxicity: Key Uncertainties and Research Needs

There is vast literature on the developmental neurotoxicity of environmental chemicals. The hypothesis of a relationship between chemical exposures and neurobehavioral changes was first raised by the evidence that lead exposure was toxic for brain development [11,12,13,14]; similar results were later identified for the exposure to other environmental contaminants in the early phase of neurodevelopment. Significant alterations in neuropsychological maturation in relation to developmental exposure to chemicals have been described for heavy metals [15,16], different classes of pesticides [17,18,19,20], polychlorobiphenyls (PCB) [21], polybrominated diphenyl ethers (PBDE) [22], and phthalates [23]. Chemicals can have an effect at any time in the process of brain development, however, the earlier the stage of brain development the greater will be the impact on brain structure and function [24].

Recently, Grandjean and Landrigan [25] performed an extensive review of studies published from 2006–2012 on the neurotoxic effects of industrial chemicals in human beings. This article updates the previous one published from the same authors in 2006 [26], where a series of 201 chemicals among metals and inorganic compounds, organic solvents, pesticides, and other organic substances were identified as neurotoxic for adult individuals, though, at that moment, only very few of such chemicals had been classified as neurodevelopmental toxicants. In particular, in the 2006 survey, lead, methylmercury, toluene and PCB were implicated in neurobehavioral deficits in children following prenatal exposures at concentrations considered as subtoxic in adults.

Since 2006, new data have emerged about the vulnerability of the developing brain and the neurotoxicity of industrial chemicals, and the recent review from the same authors reports new evidence that derives from prospective epidemiological birth cohort studies. Six additional agents have emerged as neurodevelopmental toxicants of concern. In particular, epidemiological data associate manganese, fluoride, chlorpyrifos, dichlorodiphenyltrichloroethane, tetrachloroethylene, and PBDEs with diminished intellectual functioning, learning disabilities, attention problems, aggressiveness, hyperactivity, ADHD and ASD [27,28,29,30,31]. Other suspected developmental neurotoxicants are further indicated: among these, phthalates and bisphenol A, air pollution, and comparable complex emission such as the traffic-related pollution. In particular, carbon monoxide, nitrogen oxides, and polycyclic aromatic hydrocarbons are recently reported to act as neurotoxic agents that can cause cognitive and neurological impairment [20,32], as well as ASD [33], and ADHD [34].

In spite of the impressive number of epidemiological data that indicate an inverse association between chemical exposure and child neurodevelopment (see also [35] for an updated review), there are still important knowledge gaps in this field that hamper the proper evaluation of neurobehavioral effects by risk assessors. In this framework, Bellinger [36] elegantly discussed issues concerning the validity, precision, and interpretation of epidemiologic studies of neurotoxicity. He pointed to the estimation of the dose-effect relationship as a crucial point for establishing the causative role of environmental chemicals in human diseases. Dose-effect estimation requires linking environmental exposure to the biologically effective dose of xenobiotics at the main target tissues, and then relating internal dose at target tissue with the physiological perturbation/health outcome observed. This implies both identification of reliable biomarkers of exposure (*i.e.*, peripheral indicators predictive of concentrations at target tissues) and knowledge of the mechanisms of neurotoxicity for the chemical compounds in study. Indeed, a critical gap relates to the mechanisms by which different classes of chemicals act on behavioral development. Mechanistic studies carried out in animals and in *in vitro* models point to multiple pathways and targets of toxicity for several established neurotoxicants. Chemicals may directly interfere with formation and closure of the neural tube, cell proliferation, migration, death, or synapse formation [37] inducing changes ranging from overt morphological alterations [38] to subtle dysfunction in synaptic connectivity [39]. The same chemical compound may affect different developmental processes or different cell types, depending on the time window of exposure [40]. Furthermore, compounds belonging to the same chemical class may have dissimilar mechanisms of action, as is the case for different organophosphate insecticides that produce disparate neurotoxic outcomes despite their shared property as cholinesterase inhibitors [41]. To complicate the picture further, the same chemical may have multiple mechanisms of action: agents endowed with endocrine disrupting activity may affect neurobehavioral development by directly interacting with steroid receptors in brain cells and/or in periphery, and at the same time influence the density of synaptic connections in specific brain areas with mechanisms possibly independent from their hormone-like action [42]. Thus, more experimental research is needed to elucidate the causal links between chemical exposure and disease, addressing different levels of biological organization through the combined use of *in silico*, *in vitro* and *in vivo* models. This will support building adverse outcome pathways for neurodevelopmental effects [43,44].

Even if the precision of dose estimates can be improved, a second main issue is how to capture the individual dimension of the exposure history. Different factors may indeed contribute to variability in the measured outcome, including the temporal dimension of the exposure, the co-exposure to other contaminants or stressors, and the existence of genetic vulnerability that may render an individual more susceptible than others. In addition, chemical exposure may be associated with other risk factors that should be precisely measured to prevent underestimation or overestimation of toxicant effects [45]. These factors are usually taken into account as confounders or effect modifiers in the result interpretation: among them, the most frequently considered are child sex and age, and SES indicators, whose association with neurocognitive outcomes (the higher the SES, the better the outcome) has been widely demonstrated (for a review on the effect of SES on the neurocognitive performance see [46]). Together with their role as confounders or effect modifiers with respect to toxicant exposure evaluation, these factors can have a direct beneficial or harmful effect on neurodevelopment that should be studied *per se*. For example, SES indicators, including education and/or income of either child’s parents or the community have been shown to contribute to ASD prevalence—the higher the “community” SES, the higher the probability of ASD diagnosis for children affected, especially for mild cases [47], and to ASD risk—the lower the family income, the higher the risk of ASD [48].

A third critical question, which is of particular significance for risk assessment, is that of the robustness of the outcome measurements. Developmental neurotoxicity is often evaluated by behavioral symptoms that, by their very nature, are endowed with a wide variability range and high sensitivity to both individual history (family, SES, nutritional factors) and characteristics of the neuropsychological test applied. As a matter of fact, most of prospective epidemiologic studies on neurotoxicity do not report clinically defined conditions (*i.e.*, autism or learning disability) but rather atypical behavioral traits that range from increased/decreased anxiety and aggressiveness, to poorer motor or intellectual development in a significant proportion of exposed infants/children [49]. Whether these sub-clinical behavioral alterations may signal increased risk to develop a frank neuropsychiatric disorder at some point in the individual’s life course remains to be determined. As Bellinger [50] pointed out, the characteristics of the effect outcome in brain/mental diseases has generally led to under-estimation of health risk as far as a disease-oriented approach rather than a population-oriented approach is preferred. Even a small shift in the mean IQ score in a population will result in a substantial increase in the percentage of individuals with extremely low scores, with a significant impact on economic and health costs [51].

A discussion on the methodological and statistical tools that could improve evaluation of population health burden associated with environmental contamination is beyond the scope of the present review. The harmonization of evaluation tools (*i.e.*, use of validated neuropsychological test batteries to compare the outcomes coming from different cohorts) and proper consideration of confounders would undoubtedly strengthen the robustness of human data in support of a causal association between environmental chemicals and brain diseases. However, the complex etiology of major chronic diseases, including neurodevelopmental and neurodegenerative diseases, requires innovative study paradigms and multifaceted and multidisciplinary approaches, taking into account biological plausibility as supported by experimental data, exposure and health effect modification due to intrinsic (such as genetic susceptibility) and extrinsic (such as socio-economic status) factors.

## 3. Facing Complexity: The Exposome Concept

According to the need to generate integrative models for the study of the etiology of complex diseases, the concept of the “exposome” represents the actual synthesis of this approach. It was first proposed by Wild [52] to encompass the totality of human environmental exposures from conception onwards and was developed to draw attention to the need for more complete environmental exposure data, with the aim of complementing the genome with a more comprehensive description of lifelong exposure history. Initially conceived to describe the multifactorial origin of cancer and other chronic diseases, the exposome approach may be a powerful tool also to unravel the genetic and environmental contributions to the etiology of NDDs and neurodegenerative disorders.

**Figure 1:**
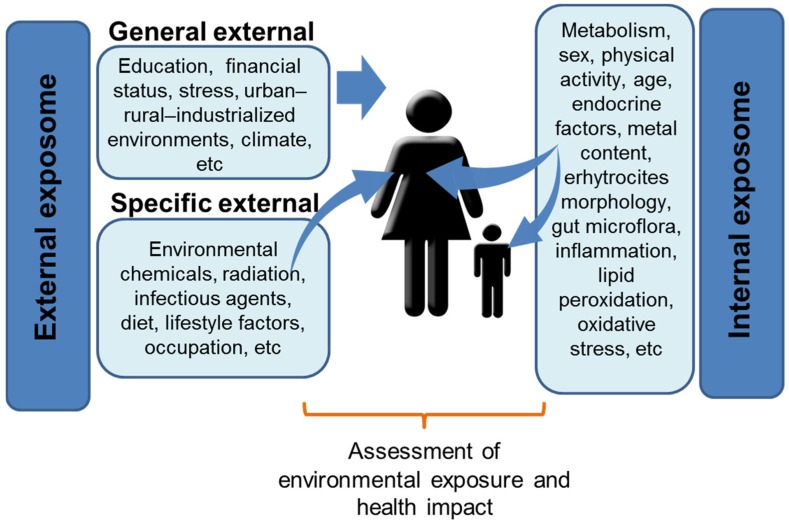
The domains of the exposome: different domains (external and internal) are illustrated. Omic signatures of the exposome can be assessed at different developmental stages in the exposed individuals to fully estimate the health impact.

The exposome paradigm analyzes both endogenous and exogenous sources of non-genetic exposure (Figure 1). In addition to environmental pollutants, the exposome includes diet, life style factors, occupation, pre-existing diseases, infections, psychological stress, and socioeconomic status, together with several endogenous factors—inflammation, hormones, metabolism, and oxidative stress—potentially influencing the response of the organism to the external factors. The overlap and the interplay between the internal and the external domains, together with their relative temporal variation, represent the most challenging features for the exposome characterization. Measuring the exposome requires a many-sided and interdisciplinary approach: the collection of data on several chemical and physical external exposures must be complemented by the characterization of downstream biological events causally linked to exposures by exploiting high-throughput omics technologies [53]. The contributions of omics techniques lie in their potential to measure “signatures” of the biological response to cumulative exposure, thus hopefully allowing a more holistic evaluation of exposure-related health effects [54].

Altogether, the exposome concept tries to overcome the paradigm of “nature *versus* nurture” and adopt one defined by complex and dynamic interactions between DNA sequence, epigenetic DNA modifications, gene expression and environmental factors that all combine to influence disease phenotypes. The European Commission launched in 2012 the Exposome European Initiative to support the development of this complex and innovative approach, and three large collaborative projects (HELIX, EXPOsOMICS and HEALS) have been funded and are under way [55,56,57]. HEALS (Health and Environment-wide Associations based on Large population Surveys) in particular comprises re-examination of data from existing large EU cohort studies on chemical exposure and neurodevelopmental disorders, to identify major knowledge gaps and select the most appropriate tools to apply the exposomic approach in a future multi-centered birth cohort study.

## 4. Multifactorial Origin of NDDs: The ASD and ADHD Examples

NDDs encompass a group of clinical heterogeneous conditions with onset in the developmental period. These disorders typically manifest early in development and are characterized by developmental deficits that produce lifetime impairments of personal, social, academic, or occupational functioning. The range of developmental deficits varies from very specific limitations of learning or control of executive functions to global impairments of social skills or intelligence. Commonly known NDDs include ASD, ADHD, communication, speech and language disorders, and genetic disorders such as Fragile X or Rett syndrome. To date, the etiological bases of the majority of these conditions are still unknown, though a great body of data supports their polygenic and multifactorial etiology [58]. In NDDs, a number of variations (*i.e.*, single nucleotide polymorphisms, mutations, deletions and copy number variants) in different genes may confer higher vulnerability to different kinds of adverse environmental factors (maternal infections, obstetric complication, and exposure to drugs or neurotoxicants during pregnancy) [59].

### 4.1. Etiology of ASD: The Environment Contribution

Genetic studies have revealed that some NDDs have a strong genetic component [58,60]; in particular for ASD, linkage studies identified many candidate genes at multiple loci on chromosomes 7q, 15q and 16p [58,61]. More recently, whole-exome analyses in sporadic cases of autism have focused on rare *de novo* mutations [62] and copy number variants linked to autism [63]. Many of the genes involved codify for proteins involved in synapse function, gene expression regulation and neural development [64]. Twin studies reported over 60% concordance for classic autism in monozygotic (MZ) twins confirming genetic heritability but also supporting the contribution of epigenetic and environmental factors to the variable expression of autism-related traits in MZ. The significant role of genes in ASD is also supported by the large difference in concordance rates between monozygotic and dizygotic twins [65,66]. However, dizygotic twins show a greater concordance than non-twin siblings [67], suggesting that a large component of the risk of autism is associated to the shared uterine environment rather than to genetics alone [65].

The hypothesis of an environmental contribution to ASD etiology originates from evidence showing a substantial increase of autism cases in a cohort of children with congenital rubella [68], and significant proportion of ASD diagnosis in children prenatally exposed to either thalidomide or valproic acid in pregnancy [69,70], or after maternal infection with cytomegalovirus [71]. Recent studies support the view that the rapid increase in ASD prevalence observed over the past few decades in US and other countries [72,73] cannot be fully accounted for on the basis of improved diagnosis, changes in classification criteria or increased social and professional awareness leading to better referral aptitudes and improved care services for ASD [74]. Thus, much research has flourished to investigate the role of environmental factors in ASD, including parental [75] and grand paternal age [76], interpregnancy interval [77], maternal infections during pregnancy [78], maternal exposure to air pollution [79,80], and environmental toxicants [81].

In 2002, a very large epidemiological study named Childhood Autism Risk from Genetics and the Environment (CHARGE) started in the US to understand the contribution of a wide spectrum of environmental contaminants to genetic vulnerability to autism (CHARGE 2006). For the first time, a study was designed to identify the specific developmental toxicants associated to autism, such as pesticides, metals, persistent pollutants and infections, and their interaction with genes, in order to understand the causes and reduce the incidence of autism. In the CHARGE study populations, the levels of these xenobiotics in blood, urine and hair specimens were analyzed, through characterizations of metabolic, immunologic and genetic polymorphisms [82]. So far, the results of CHARGE indicate a significant association of ASD risk with residential proximity to freeways [83] prenatal exposure to agricultural pesticides [organophosphates (OPs) and pyrethroids] [84], and maternal fever during pregnancy [85]. On the contrary, periconceptional folic acid and vitamin supplementation during pregnancy appear to be associated to a lower risk of ASD, at least for mothers (and children) with specific genetic susceptibility (e.g., *MTFHR* 677 TT or *CBS* rs234715 GT + TT) [86,87]. Although remarkable for the great effort to collect information on a wide range of environmental factors, CHARGE presents some limitations, mainly due to the fact that it is a case-control study. CHARGE enrolls children between the ages of 24–60 months (2–5 years): cognitive and social development is directly assessed, and current level of toxicants is measured on biological specimen collected on participants. On the contrary, information on demographics and environmental exposure *sensu lato* (mother’s medical, reproductive and pregnancy history; child’s illnesses and medications; metals, pesticides and household product use; maternal lifestyle; diet and residential history; parental education and occupation) is gathered retrospectively, through the completion of specific questionnaires. Thus, while diagnosis for the definition of cases and controls is accurate, information on life-long exposure to environmental factors is potentially affected by recall bias, leading to differential misclassification of exposure (much more relevant as larger the distance between recall and event is). This can limit the validity of the assessment of environmental risk factors role on neurobehavioral development and of their effect sizes that need to be confirmed in large birth cohort studies.

### 4.2. Etiology of ADHD: The Environment Contribution

ADHD is a common heterogeneous neuropsychiatric disorder characterized by debilitating social and behavioral symptoms of excessive inattention, hyperactivity and impulsivity [88,89]. Many genetic studies indicate that ADHD has a strong heritability, also related to a complex multigenic etiology [90,91] consistent with small effects due to multiple genes. Pathogenetic models of ADHD have traditionally focused on many candidate genes involved in neurotransmission and catecholamine synaptic dysfunction [92,93]. The most robust evidence of association with ADHD has been shown for dopamine receptors DRD4 and DRD5 variants, for dopamine transporters gene *DAT1* (*SLC6A3*) regulating the reuptake of dopamine in the presynaptic cleft [94], and for synaptosomal protein SNAP-25 [95]. Several polymorphisms in ADHD candidate genes were identified, such as the valine/methionine of the gene encoding catechol-*O*-methyltransferase (COMT), which affects the catalytic activity of the enzyme for the degradation of dopamine, and the C102T polymorphism of the serotonin receptor gene *5-HT2A* [96].

Recent data suggest that after genetic factors, the association between ADHD and prenatal exposure to several environmental factors is substantial. In particular, there are many environmental risk factors which may moderate the strong genetic risk for ADHD or ADHD symptoms (attention problems, impulsivity and hyperactivity), such as prenatal exposure to nicotine [97], alcohol [98], drugs [99,100], PCB [101,102], heavy metals such as lead (Pb) [103,104,105] and mercury (Hg) [106,107], organophosphate pesticides [17,27,108], maternal stress in pregnancy [109,110], low birth weight and prematurity [111,112].

Polanska and coworkers performed an exhaustive review of recent studies assessing the role played in ADHD etiology by environmental pollutants including polycyclic aromatic hydrocarbons, phthalates, polyfluoroalkyl chemicals, BPA, tobacco and alcohol [113]. The review shows that although much research has been performed on this topic, results are still largely inconsistent. Specifically, the same chemical/exposure factor can be significantly associated to ADHD-related symptoms in some studies and not in others: for example, maternal smoking is significantly associated with ADHD in eight out of 15 studies. Moreover, within the same studied population, a given chemical/exposure factor can be significantly associated only to some of the symptoms characterizing ADHD, as in the case of phthalates. Examination of the main characteristics of the 40 studies reviewed (studied population, type of study, definition of the exposure, test used, and confounders) revealed many critical points already discussed in the previous section. Overall, the studies included in the analysis, either cohort, cross-sectional, or case-control, performed only one single measurement of exposure or in many cases reconstructed the exposure by interviews or retrospective reports, thus implying the risk of a significant recall bias. Only few studies (*i.e.*, those concerning phthalates and bisphenol A) measured exposure by means of chemical metabolites in biological matrices such as cord blood or urine (in mother and child). Furthermore, the studies examined in the Polanska's review were heterogeneous as for confounders included in the statistical analyses (age, sex, breastfeeding, stress during pregnancy, life styles, ethnicity, SES, *etc*.), instruments used for case classification, and behavior assessment. All these limitations stress, as in the case of the CHARGE study conducted on ASD, the importance to carry out comparable prospective birth cohort studies, controlling for the same potential confounders, collecting exposure measurement to reflect cumulative exposure or exposure at sensitive developmental periods, and evaluating genetic makeup.

## 5. Gene Polymorphisms as a Source of Vulnerability

Among the potential sources of inter-individual variation in susceptibility to chemicals in population studies are genetic polymorphisms. Common gene variants may induce susceptibility to environmental factors by increasing or decreasing physiological responses to common effects from intrauterine infections and cytokines, or from environmental toxins, through the mother’s internal or external environment [114]. Single Nucleotide Polymorphisms (SNPs), namely variations at a single position in a DNA sequence among individuals, are the most common type of human genetic variation. In the past decade, much research has been devoted to find SNPs that may help predict an individual’s response to certain drugs, susceptibility to environmental pollutants, and risk of developing particular diseases [115]. In particular, it is suggested that variations in a group of so called “environmental responsive genes” may confer higher vulnerability to the adverse effects of environmental toxicants [116]. Notably, several studies have identified SNPs in genes involved in the detoxification of environmental pollutants in some individuals with ASD, and it is estimated that more than 100 such genes may contribute to ASD risk [117]. The number of studies investigating the role played by genetic polymorphisms in modifying neurotoxicity has increased rapidly in recent years, but the results of these epidemiologic studies are far from being consistent. In the following two paragraphs, we will review the most recent data investigating common polymorphisms as a source of variation in susceptibility to the neurotoxic effects of Hg and OPs in children.

### 5.1. Gene Polymorphisms Increase Susceptibility to Mercury

Hg is one of the most well studied environmental pollutants. Methylmercury (MeHg) originates from methylation of inorganic Hg by bacteria in aquatic systems [118], it is absorbed from the human gastrointestinal tract and readily crosses the placenta and blood-brain barrier [119]. The intake of seafood is the main route of human exposure to MeHg [120]; seafood contains many important nutrients for brain maturation such as long chain polyunsatured fatty acids (PUFAs) which are the major benefits of fish-derived lipids [121]. However several studies have demonstrated that the increase of fish consumption during pregnancy is responsible of several adverse effects of prenatal exposure to MeHg [122], but the results of the epidemiological studies conducted among fish-consumer populations remain controversial. In a cohort of the Faroe Islands, elevated cord blood Hg levels were significantly associated with adverse effects on motor, attention and language functions in children at seven and 14 years of age [123,124]. On the contrary, three cohort studies conducted in the Seychelles Islands, where prenatal exposure to MeHg occurs at one of the highest levels in the world [125,126,127], did not find an association between elevated Hg levels and neurodevelopment functions. A population-based birth cohort from Spain was studied at four years of age, showing that Hg levels in hair and children’s motor and cognitive abilities were inversely related [128]. In contrast, the results of a prospective birth cohort study in Northern Italy did not indicate any correlation between prenatal Hg exposure and child neurodevelopment [129]. These conflicting findings have been related to variations in genes implicated in MeHg uptake, distribution and excretion [130,131] and/or to the beneficial effects of PUFAs present in fish, which play a protective role in brain development [132,133].

The hypothesis that genetic factors may increase susceptibility to MeHg toxicity has been addressed by several epidemiological studies, by considering gene mutations that affect the absorption, distribution, metabolism and elimination of elemental Hg and MeHg in the body. Echevarria and coworkers [134] identified a SNP in the exon 4 of the gene encoding the heme biosynthetic pathway enzyme coproporphyrinase oxidase (CPOX), which may be responsible for susceptibility to the neurobehavioral effects associated to elemental Hg exposure in humans. Woods and coworkers then analyzed the interaction between this same SNP and Hg exposure through dental amalgam tooth filling on neurobehavioral functions of children aged eight to 12 years. The allele frequencies was equally distributed among males and females (about 15%), but Hg exposure was strongly associated with a reduction in cognitive performance in males carrying the *CPOX4* allele [135]. The mechanisms by which *COPX4* allele confers higher vulnerability to Hg exposure are not clear: the heme biosynthetic pathway plays a regulatory role in neural and synaptic development, and it is possible that both Hg and the genetic variant impact on this same pathway with additive effects on neurodevelopment [136]. Notably, children with ASD have higher mean urinary porphyrin concentrations, suggesting that the processes that change porphyrin excretion might be related with processes involved in ASD pathogenesis [81]. Other studies have described Hg effects on neurodevelopment in boys with genetic variants of metallothionein (MTs) proteins that play a crucial role in both dispersal and storage of metals such as Hg in the body [137]. The MTs display two different isoforms in humans: MT1M and MT2A, which can modify the Hg toxicokinetics in adults [138], and possibly enhance the adverse neurobehavioral effects of MeHg exposure in children. Woods and coworkers [139] showed that both variants exacerbated the Hg effects on neurological functions in children, in particular for verbal learning, memory and executive functions. These findings are consistent with those of a mouse study where the deletions of *MT1/2* genes worsen learning and memory impairments associated with Hg exposure [140].

In a prospective study, Ng and coworkers [141] examined the interaction between different alleles of the apolipoprotein E and neurodevelopment in two-year-old children exposed to Hg as measured in cord blood. The gene product, apolipoprotein E, is a protein transporter expressed in the brain; the *Epsilon4* allele, considered a risk factor for Alzheimer disease, is associated with poor neural repair function and it has also been shown to modulate the neurobehavioral toxicity of lead in adults [142]. The authors reported that the individuals carrying the *Epsilon4* allele are more vulnerable to the adverse effects of Hg on neurodevelopment than individuals carrying other alleles [141].

Another line of evidence reported variations in genes implicated in the placental transfer of xenobiotics: MeHg is able to cross the placenta through specific transporter proteins, the superfamily of ATP binding cassette (ABC) transporters, a widely expressed protein family responsible for the active transport of various xenobiotics across the cell membranes. Llop and coworkers [143] evaluated the relationship between some polymorphisms in *ABC* genes (*ABCB1*, *ABCC1* and *ABCC2*) and prenatal exposure to MeHg in two Mediterranean birth cohorts. The results showed that the association between maternal fish intake and mercury in cord blood depends on the child’s genotype for ABC transporters, as risk alleles cause increased accumulation of MeHg during early development.

Recently, the Avon Longitudinal Study of Parents and Children (Bristol, UK) analyzed the association between prenatal MeHg exposure and IQ scores at eight years in 1135 children for whom data on 247 SNPs within relevant genes were available. Among 40 SNPs showing nominally significant main effects, MeHg interactions with IQ scores were detected for paraoxonase 1 (*PON1*), progesterone receptor, transferrin and brain-derived neurotrophic factor. Thus, heterogeneities in several relevant genes not specifically linked to Hg uptake or excretion indicate possible genetic predisposition to Hg neurotoxicity in a substantial proportion of a population with a low level of Hg exposure [45]. Finally, a very recent study carried out by Woods and coworkers [144] chose 13 candidate genes identified as associated with various neuropsychiatric disorders and also with alterations in Hg toxicokinetics and tissue distributions [144]. In particular, these genes present different variants that modify the effects of Hg exposure on a broad or limited range of neurobehavioral functions in children. Of the 13 genes evaluated, only four of them showed variants that significantly alter the effects of Hg exposure on neuropsychological domains in children: the gene encoding the heme pathway enzyme, *CPOX4*, the *MTs* with their two isoforms *MT1M* and *MT2A* and the cathecol-*O*-methyltransferase gene (*COMT*) that has been linked to diverse neuropsychiatric conditions such as schizophrenia and ADHD.

In conclusion, the observations raised by different epidemiological studies seem to support the hypothesis that some genetic variants may be in part responsible for the increased susceptibility of the negative effects of Hg exposure on neurobehavior in children (Table 1). Regression coefficients β of the most relevant effects reported in the table provide information on the effect size of the studies. It should be kept in mind that regression coefficients β represent the change in the dependent variable (e.g., neuropsychological outcome) for any unitary increase of the independent variable (e.g., blood levels of Hg). As an example we refer to paper [44]: in subjects bearing TT allele in TF gene, an increase of one unit in the Log_10_ cord blood Hg (*i.e.*, a ten-fold increase in the cord blood Hg value) yields a mean decrease of 22.7 points in the WISC-III Performance IQ score, that can be relevant since the WISC-III Performance IQ score in typical developing children has a mean value of 100, and borderline functioning can be suspected below the score of 84.

Besides the genetic polymorphisms modulating Hg toxicity, the social environment, the potential for MeHg to interact with other chemicals present in marine food and the protective effects of some nutrients present in fish (e.g., PUFA, selenium) might also significantly contribute to the final behavioral outcome [145]. In addition, we must consider that the clinical significance of a gene-environment interaction at the population level depends not only on the size of the effect of a genetic variant on toxicity, but also on the frequency of that variant in the population [146]. Hence, the relevance of a gene-environment interaction on vulnerability must be always evaluated with respect to the specific population under study, taking into account the size of the genetic effect on environmental toxicity together with the frequency of that genetic subgroup in that population. Distribution of genetic variants is known only for the populations where specific epidemiological studies have been conducted, and differs across populations: for this reason, the information on the distribution of a genetic trait available in one population cannot be translated to others, making the plain extrapolation of toxicity effects due to gene-environment interaction across populations questionable.

### 5.2. Vulnerability to Organophosphate Pesticides and Paraoxonase 1

OPs are a large family of non-persistent chemical compounds used for the elimination of insects in agriculture and for residential use. Their toxicological mode of action is primarily associated with their capability to inhibit the enzyme acetylcholinesterase (AChE), thus preventing the degradation of the neurotransmitter acetylcholine and consequently increasing both its concentration and permanence in the synapse [147]. Several studies have demonstrated that developmental exposures to OP pesticides can be toxic for both humans and animals [148,149] even at doses below those that inhibit brain AChE. OPs are able to interfere with neurotransmitters, neural development [150] and synapse formation [151] and behavioral development [152]. In humans, prenatal exposure to OPs was negatively associated with abnormal reflexes [153], smaller head circumference [154], lower IQ and increased risk of neurodevelopmental delay in childhood [17,29,31,155].

**Table 1:** Genetic polymorphisms and Hg exposure in humans.

Single nucleotid polymorphism (SNP)	Hg source	Size of the study (age)	β regression coefficient	Ref
ATP Binding Cassette (*ABC*) transporter genes	Environmental exposure (fish diet)	1651 (birth cohort)	Interaction Log_2_ fish intake*genotype on log_2_ cord blood MeHg	[143]
			rs2032582: TT vs GG β = −0.49 (CI = −0.71 to −0.26)	
			rs11075290: TT vs CC β = −0.28 (CI = −0.51 to −0.06)	
			rs2273697: GA+AA vs GG β = 0.16 (CI = 0.01 to 0.32)	
Trasferrin (*TF*), Brain Derived Neurotrophic Factor (*BDNF*)	Not known (Avon longitudinal study of parents and children)	1135 (0–8y)	Log_10_ cord blood MeHg on WISC-III Performance IQ in selected genotypes	[45]
			*TF* rs3811647: AA β = −22.7 (CI = −44.0 to −1.5)	
			*BDNF* rs2049046: AA β = −13.7 (CI = −26.9 to −0.4)	
Metallothionein (*MT1M*, *MT2A*)	Dental amalgalm tooth filling	120 boys, 118 girls (8–12y)	Log_e_ urinary Hg on RAVLT8 in selected genotypes	[139]
			*MT1M* rs2270837: GA+AA β = −2.11 (SE = 0.74)	
			*MT2A* rs10636: GC+CC β = −1.96 (SE = 0.86)	
			Log_e_ urinary Hg on Visual Spatial—Digit Symbol in selected genotypes	
			*MT2A* rs10636: GC+CC β = −12.9 (SE = 4.77)	
Glutathione related genes: Glutamyl-cysteine-ligases modifier subunit (*GCLM*) Glutathione-*S*-transferase μ1 (*GSTM1*)	Environmental exposure (fish diet)	400 (adults)	Genotype on Log_e_ blood Hg	[156]
			*GCLM*-588: TT β = −0.32 (p = 0.017)	
			GSTM1*0: Homozygous β = 0.20 (p = 0.017)	
			Genotype on Log_e_ hair Hg	
			*GCLM*-588: TT β = −0.33 (p = 0.009)	
			GSTM1*0: Homozygous β = 0.20 (p = 0.013)	
Apolipoprotein E (*APOE*)	Environmental exposure (fish diet)	180 (0–2y)	Log_e_ cord blood MeHg on CDIIT cognition score in selected genotypes	[141]
			*APOE*: ε4 carrier β = −8.47 (CI = −16.1 to −0.84)	
			Log_e_ cord blood MeHg on CDIIT social score in selected genotypes	
			*APOE*: ε4 carrier β = −11.02 (CI = −20.85 to −1.19)	
			Log_e_ cord blood MeHg on CDIIT whole test score in selected genotypes	
			*APOE*: ε4 carrier β = −10.45 (CI = −17.36 to −3.54)	
Coproporphyrinogen oxidase (*CPOX*); Metallothionine (*MT1M*, *MT2A*); Catechol-*O*-methyltransferase (*COMT*); Tryptophan 2,3-dioxygenase (*TDO2*)	Dental amalgalm tooth filling	120 boys, 118 girls (8–12 y)	Log_e_ urinary Hg on neurobehavioral tests in selected genotypes (boys)	[144]
			*CPOX* rs1131857: AC+CC	
			Attention: β range = −2.45 (SE = 0.97) to −18.1 (SE = 4.89)	
			Visual-Spatial: β = −20.7 (SE = 6.08)	
			Executive function: β = 22.26 (SE = 7.03)	
			Learning & Memory: β range = −1.78 (SE = 0.80) to −2.24 (SE = 0.82)	
			Motor: β range = −3.46 (SE = 1.65) to −7.16 (SE = 2.9)	
			*MT1M* rs2270837: GA+AA	
			Learning & Memory: β range = −1.56 (SE = 0.53) to −2.11 (SE = 0.74)	
			*MT2A* rs10636: GC+CC	
			Visual-Spatial: β range = −8.21 (SE = 2.62) to −12.9 (SE = 4.77)	
			Learning & Memory: β range = −1.96 (SE = 0.86) to −6.49 (SE = 2.72)	
			*COMT* rs4680: GA+AA	
			Attention: β = −5.05 (SE = 1.95)	
			Visual-Spatial: β range = −16.49 (SE = 5.52) to −32.46 (SE = 11.28)	
			*COMT* rs4633: CT+TT	
			Attention: β range = −4.58 (SE = 1.84) to −13.79 (SE = 6.23)	
			Visual-Spatial: β range = −16.19 (SE = 5.53) to −35.71 (SE = 10.87)	
			*COMT* rs6269: AG+GG	
			Visual-Spatial: β range = −8.48 (SE = 3.83) to −17.92 (SE = 6.8)	
			*TDO2* rs3755907: GA+AA	
			Attention: β = −14.66 (SE = 7.12)	
			Visual-Spatial: β = −0.16 (SE = 0.06)	
			Motor: β = −9.36 (SE = 3.90)	

CI: confidential interval; WISC III: Wechsler Intelligence Scale for Children III^rd^ version; RAVLT8: Rey Auditory Verbal Learning Test 8; CDIIT: Comprehensive Developmental Inventory for Infants and Toddlers.

A key role in OP metabolism and detoxification pathways is played by the PON1 enzyme [157], which metabolizes the activated form of OP pesticides [158]. *PON1* gene presents different genetic polymorphisms that can facilitate or delay the metabolic inactivation of active compounds [159,160,161]. Several studies have characterized the functional polymorphisms of PON such as *PON1_162_*, *PON1_108_*, *PON1_55_*, *PON1_192_* [162]. In particular, the most studied is the PON1_192Q_ alloform, which is associated to a missense mutation in the coding region of *PON1* gene that determines the glutamine (Q)/arginine (R) substitution at codon 192: this form hydrolyzes OP oxons as chlorpyrifos-oxon and paraoxon more efficiently than does *PON1_R192_* [163] conferring a greater degree of protection from OP exposure [162]. The second polymorphism is associated with a missense mutation in the promoter region of the *PON1* gene, which determines the leucine (L)/methionine (M) at position 55 with PON1_M55_ being associated with low plasma PON1. In 2003, Costa and coworkers identified two other members of the *PON* family: *PON2* and *PON3*, which are located in the long arm of the human chromosome 7 (q21.22). *PON1* has genetic variants differently expressed in diverse ethnic groups, as the frequency of the *PON1_Q192_* isoform is higher in Caucasians than in both Mexicans and African-Americans [164]. *PON1* expression in humans is also age-dependent, as newborns have PON1 levels much lower than adults [164,165] and this expression increases steeply from six months to two years of age, continuing to increase until seven years of age [166]. In addition, children carrying the alleles *PON1_R192_* and *PON1_C108_* have higher activity of the enzyme than children with *PON1_Q192_* and *PON_T108_* [167].

The first evidence of a potential effect of *PON1* gene variants on neurodevelopment after OPs exposure was suggested by Berkowitz and coworkers [154], who analyzed the relationship between pesticide metabolites and birth outcome. They observed only a trend toward smaller head circumference in children with mothers with low PON1 activity and high OP metabolites levels in blood. As small head size has been found to be predictive of subsequent cognitive ability in children, the authors advanced the hypothesis that *PON1* polymorphisms might be implicated in the adverse OP effects on neurodevelopment [154]. In 2005, D’Amelio and coworkers assessed linkage/association between autism and PON1 variants. Three functional SNPs, *PON1 C-108T*, *L55M*, and *Q192R*, were assessed in 177 Italian and 107 Caucasian-American first degree relatives of primary autistic probands. Interestingly, Caucasian-American and not Italian families displayed a significant association between autism and *PON1* variants less active *in vitro* on the OP diazinon (R192) [168]. Other studies have evaluated the associations between PON1 activity, OP pesticides and neurodevelopment. In particular, Engel and coworkers in a New York birth cohort found an association between specific metabolites indicative of prenatal exposure to pesticides and the increase in abnormal reflexes in newborns whose mothers had lower levels of *PON1* expression [153]. In a subsequent study carried out in the same cohort of 404 children, maternal urinary levels of dialkylphosphates (DAPs), representative of total OP exposure, were negatively associated to neurodevelopment at both 12 and 24 months and six to nine years of age, especially for children whose mothers showed the *PON1_192Q_* genotype [29]. Eskenazi and coworkers (2010) analyzed the association between *PON1_−108_* genotype and mental development scores of children, showing that there is a stronger association between children with *PON1_−108T_* allele who display lower scores on neurodevelopmental indexes and OP pesticide exposure *in utero* (as measured by maternal DAPs) [158]. The same authors have confirmed the association between maternal OP metabolites and neurobehavior in children at school-age: in the case of maternal *PON1_−108_* genotype, the association between neurobehavioral deficits and maternal DAP levels is weaker a school age than at younger age, thus indicating interaction among gene variants, chemical exposure and age of assessment [169]. Some studies have also suggested that variants in PON1 may play a role in neuropsychiatric disease such as schizophrenia [170], depression [171] and autism [172,173] with mechanisms that are far from being understood. As PON1 plays a prominent role among the enzymes that prevent or mitigate damage caused by reactive oxygen species, the reduction of its functional activity might render the developing brain more vulnerable to oxidative stress.

On the basis of the studies reported above, it appears that variants of the PON1 gene may increase vulnerability to OP effects on neurodevelopment; it is worth mentioning that lower PON1 activity or significant association with specific SNPs in *PON1* have been reported in some ASD cases, but these evidences are far from being conclusive (reviewed in [81]).

## 6. Susceptibility to Environmental Chemicals: A Key Role for Epigenetics?

Mutations or SNPs in several genes may explain differential vulnerabilities, but genes are not the only actors playing a role in health and disease. Increasing data shed light on the complex relation between genes and their expression by means of epigenetic mechanisms, and identify a further level of vulnerability for chemical exposure during neurodevelopment [174]. 

As described in Section 4, the numerous gene polymorphisms identified in specific NDDs may possibly contribute to their pathogenesis. However, monozygotic twins share nearly 100% of their genetic polymorphisms, and there is no NDD for which concordance rates between monozygotic twins reach the estimated heritability. Epigenetic mechanisms may provide a molecular link between environmental and genetic factors in the development of psychopathology [175]. As epigenetic programming determines the state of expression of genes, epigenetic differences could have the same consequences as genetic polymorphisms [176]. Moreover, there is experimental evidence that exposures during the prenatal window can influence disease risk transgenerationally through epimutations in the germline [174]. Therefore, epigenetic mechanisms may account for much of the discrepancies between monozygotic discordance rates and heritability estimates found in major NDDs [177].

Epigenetic mechanisms are related to relevant modifications in the genome that influence gene expression without changes in nucleotide sequence. These modifications alter how the histone proteins are complexed with DNA to form the chromatin, and are stable across generations [178]. The epigenetic modifications include several processes, as DNA methylation, histone modifications, different mechanisms of chromatin organizations and microRNAs [179], which have the potential to influence the long-term effects of environmental exposure to neural gene expression and therefore the risk of developing NDDs [180]. Some epidemiological studies suggest that environmental exposure to various neurotoxic chemicals, such as metals, pesticides and endocrine disrupters induce aberrant changes in epigenetic pathways with consequent adverse effects on human neurodevelopment [181].

It is known that epigenetic modifications are important tools by which neurons modify their transcriptional response to developmental and environmental factors [182] but the mechanisms by which early exposure to environmental toxins induce permanent epigenetic changes able to interfere with typical brain development are far from understood. Variations in genes encoding epigenetic regulators may play a critical role, as they hamper the capacity of neurons to respond appropriately to environmental signals in critical developmental phases [183]. Of particular interest are mutations which involve many chromatin regulators, as proteins with methylbinding domain such as the methyl CpG binding protein 2 (MeCP2) that causes Rett Syndrome. MeCP2 is a DNA binding protein that shows high affinity for methylated cytosine and acts either as a positive or negative modulator of gene expression [184]. MeCP2 exerts an important role in the regulation of synaptic plasticity [182,185]; there are many examples of epigenetic alterations linked to reduced expression of *MeCP2* and other epigenetically regulated genes such as *FMR1*, which is associated to the Fragile X syndrome, idiopathic autism, and intellectual disabilities [186]. Thus environmental toxins and genetic vulnerability (*i.e.*, variations/mutations in epigenetic regulators genes) may converge on the same epigenetic pathways, leading to phenotypic changes [187]. Environmental toxins may also directly interfere with modulation of DNA expression, as in the case of the antiepileptic agent valproic acid (VPA). As previously mentioned, VPA is one of the most studied chemical agents linked to autism, as maternal use of VPA during pregnancy is associated with a significantly increased risk of ASD and other developmental disabilities [188]. Multiple mechanisms are called upon to explain both the therapeutic and neurotoxic effects of VPA: direct interference with GABAergic neurotransmission, with folate metabolism and increased production of free radicals. The role of VPA in epigenetic regulation has only recently come to light. In particular VPA is a non-selective inhibitor of histone deacetylase of class I and II (HDAC1 and HDAC2) expressed in the brain [189]. Inhibition of HDACs is thought to occur through direct binding of VPA to the active site of the enzyme, preventing the hydrolytic removal of acetyl groups from DNA. The uncondensed chromatin may be exposed to several transcription factors that can induce excessive transcription of potentially damaging genes, such as *Bcl-2* and *Hoxa1*, thus interfering with cell proliferation, morphogenesis, programmed cell death and/or synaptogenesis depending on the time window of exposure [190].

## 7. Not in Our Genes: Socioeconomic Factors Modulate Environmental Chemicals’ Toxicity

Genes are only one among many factors modulating the response of an organism to adverse environmental stressors. Adverse maternal conditions, such as stress, infections, and malnutrition can profoundly interfere with fetal brain development through a plethora of mechanisms, including abnormal activation of the hypothalamic-pituitary-adrenal axis, neuroinflammation, alteration of the hormonal milieu, and dysfunction in the immune responses.

The observation that health and chemical burden are sustained at higher level by low SES populations informs on the potential risk for human health in those population in which cumulative risk factors, such as psychological stress due to socioeconomic deprivation and chemical exposure could occur [191,192]. In this respect, recommendations from the US National Academy of Science to the US Environmental Protection Agency stated that nonchemical stressors and other important aspects of vulnerability should be adequately incorporated into the risk assessment process [193,194]. Cumulative risk assessment represents a procedure for processing relevant information to analyze, characterize, and possibly quantify the combined harmful effects from exposure to a mixture of both chemical and nonchemical stressors. Overall, sufficient attention has been kept in considering cumulative risk which derives from exposure to chemical mixtures, though several methodological limitations have so far allowed just an estimation of the cumulative risk [195]. Few studies, however, used the “allostatic load model”, which take into account the cumulative biological burden triggered by responses to a specific “ecological niche”, and potentially includes several categories of stressors. In the allostatic load model, it is implicit that complex biological mechanisms are involved in the adaptive process defined as allostasis, which maintain homeostasis by producing multiple physiological mediators (e.g., activation of the HPA axis, sympathetic and parasympathetic regulations, and inflammatory and oxidative stress molecules). Chronic allostatic load could result in over-activity and dysregulation of allostasis mediators and determine co-occurring risk across multiple physiological systems and accumulation of such risk across time at the individual level [196]. The individual vulnerability rising from allostatic load can in turn influence responses to chemical exposure modulating response and resilience, with health effects which appear dependent on geographical, race and social factors [197].

Both epidemiological and experimental studies have explored hypotheses on the interaction between allostatic load and neurotoxicant exposure during development suggesting common target physiological systems and pathways for both toxicants and stress. In a very recent study conducted on the US cohort of women in reproductive age enrolled inside the National Health and Nutrition Examination Survey (NHANES) program, chronic stress was found to modify the association between elevated lead/methylmercury exposure and race/ethnicity [198], highlighting the importance of evaluating chemical and nonchemical stressor exposures. Previous studies had showed controversial relationships between higher environmental chemicals’ exposure and lower social economic status, suggesting that more epidemiological research is needed to clarify which variables (chemical and nonchemical) are really involved in the potential interaction between chronic stress and chemical hazard [199,200].

## 8. Unraveling Complexity: The Need for Experimental Models

The epidemiological and clinical data collected so far, while supporting the hypothesis of a substantial contribution of environmental factors to NDDs, highlight the difficulty of establishing causative links between each of these factors and the health outcome. The simultaneous exposure of an individual to multiple risk factors, which may interact in an additive, synergistic, or even antagonistic way, remains to be explained and defined, especially in a mechanistic perspective. The exposome approach has the ambition of unfolding such complexity by means of several omic signatures possibly describing exposure, effects, individual vulnerability and their dynamic interplay [53].

However, when one tries to apply the exposome concept to quantify the effects of environmental exposures on neurodevelopment, a major critical issue dampens enthusiasm: the brain, the target organ of neurotoxicants, is not accessible unless highly invasive or extremely costly (e.g., neuroimaging) methods are used. Peripheral biomarkers of exposure so far available for many environmental chemicals are indeed poor predictors of effects on the CNS [201] and estimating the concentration of a toxic compound or metabolite in the brain on the basis of concentrations found in other matrices may lead to exposure misclassification [36]. The same holds true for biomarkers of effect, which should measure early biological changes related to exposure and also predict health effects. To date no available biomarker is a clear and validated indicator of typical brain development: recent studies suggest that levels of inflammatory cytokines in amniotic fluid could predict ASD risk [202]; placental miRNA expression profiles and DNA methylation of specific genes are associated with measures of neurobehavioral outcome in the infants as well as with increased risks of neurological and neurodegenerative diseases [203]; altered amyloid-beta protein in plasma is related with neurodegenerative risk after prenatal Pb exposure [204]. It is widely acknowledged that much research is needed to identify omic biomarkers in peripheral tissues that can reliably inform on typical and “abnormal” brain development. Even if animal models can be extremely useful to validate peripheral biomarkers as informative measures also of CNS status (e.g., for oxidative stress biomarkers [205,206]) evidence is still scant on this topic.

In this framework, experimental *in vivo* models may be extremely helpful to generate mechanistic hypotheses and identify robust biomarkers of exposure, effects and susceptibility, to be subsequently verified in large prospective cohort studies. Studies with laboratory rodents permit intensive and methodological evaluation of important parameters, such as dose-response relationships, critical periods of susceptibility, and the relative contribution of genetic, epigenetic and environmental factors. Since the mid-seventies of the past century, when “behavioral teratology” originated as a sub-speciality of teratology to explain the behavioral anomalies induced by many teratogens in infants, the use of animal models has been of paramount importance to investigate the mechanisms by which chemicals influence brain development in humans [207]. In particular, animal models are a powerful tool to test what gene-environment combination or what combination of adverse environmental factors may produce significant disruption of neurobehavioral development that lead to clinically-relevant outcomes. Notably, this kind of information can only be achieved by testing the living organism as the face validity of an animal model of NDD consists primarily in a robust behavioral phenotype [208].

However, to date, the neurobehavioral toxicity of environmental pollutants and the contribution of candidate genes or adverse environmental factors to NDDs have typically been tested *in vivo* independently, with very few attempts to identify the effects of their interaction. In the following sections, we will review the *in vivo* laboratory studies analyzing the neurobehavioral effects of different environmental chemicals in combination with (i) mutations or variants in NDD candidate genes or (ii) maternal stress in pregnancy.

### 8.1. Gene Polymorphisms and Environmental Contaminants: Mouse Studies

Much information on the possible mechanisms underlying neurobehavioral toxicity of different environmental chemicals has been derived from studies on wild-type strains of laboratory rats and mice, expressing “typical” behavioral profiles. Much less is known on the potential effects of neurotoxic chemicals in the rodent models of NDDs that have been available so far. Studying the effects of developmental exposure to neurotoxicants such as heavy metals or OP insecticides in “vulnerable” animal models (*i.e.*, bearing mutation in NDD candidate genes or presenting abnormal behavioral traits) could significantly contribute to the understanding of the complex etiology of NDDs.

There are several mouse models that display face and construct validity related to some human NDDs, including cognitive impairment, deficits in both social behavior and communication and motor development [208]. In spite of the current availability of a wide number of *in vivo* models, there is a surprising paucity of data on the interaction between vulnerable genotypes or phenotypes and neurotoxic agents. This is likely due to feasibility constraints: actually cumbersome neurotoxicological experiments with developmental exposures in mouse lines that do not breed easily may be a difficult and costly task. 

Among the gene-environment interaction studies, two of them investigated the effects of developmental exposure to CPF and CPF-oxon (CPO) in reeler mice [209,210]. Reelin is a protein of the extracellular matrix with a role in neuronal migration and repeatedly implicated in ASD [211,212]; reeler mice bear a spontaneous mutation for the *reelin* gene and display neuroanatomical and behavioral alterations partly resembling those found in ASD and schizophrenic patients [24,213]. The two neurotoxicological studies in reeler mice [209,210] both indicated a paradoxical effect of mitigation of the behavioral abnormalities due to decreased reelin levels, and thus failed to identify the expected additional detrimental effects of both insults. Such a “failure”, however, could be due to specific cholinomimetic effects of the chlorpyrifos oxon and does not imply that paradoxical mitigations would be the final outcome when testing other gene-environment interactions.

PBDE neurotoxicity has been evaluated *in vivo* in a mouse model of Rett Syndrome, a neurodevelopmental disorder resulting from mutation of the *MeCP2* gene. In this study, *Mecp2-308* mutant mice (bearing a Mecp2 truncated protein at Aa 308) were prenatally exposed to human-relevant doses of PBDE. A rather complex pattern of effects was evident [135]: (i) prenatal PBDE exposure resulted in long-lasting effect on global DNA methylation, cognitive and social behaviors primarily in the female offspring (in both *Mecp2* heterozygous and wt); (ii) a synergistic detrimental interaction of PBDE exposure and *MeCP2* mutation was observed in long-term spatial memory.

In DISC1 mice, a mouse model of schizophrenia, chronic lead exposure exacerbated the behavioral alterations typical of these mutant mice, exaggerated responses to the NMDAR antagonist, mildly impaired pre-pulse inhibition of the acoustic startle, and also enlarged lateral ventricles. These findings support a new mechanistic pathway through which lead may contribute to the pathogenesis of neurodevelopmental diseases in susceptible individuals [214].

Another line of research concerns the interaction between gene polymorphisms implicated in the efficiency of detoxification or metabolic pathways, and chemical exposure in genetically-modified mouse lines. Among the OPs, special attention has been devoted to chlorpyrifos (CPF) and/or chlorpyrifos oxon (CPO), as several experimental data have indicated that CPF has significant behavioral toxicity (for review see [215]). CPF and other OP compounds primarily acts as AChE inhibitors leading to overstimulation of cholinergic synapses; after adsorption, CPF is metabolized in the liver by cytochrome P450 enzymes that desulfurate CPF to the active metabolite CPO. As described above, epidemiological data indicated the importance of investigating the role of the OP detoxification enzyme PON1 and its alloforms, to explain increased OP vulnerability. Of particular relevance appears the common *PON1_R192Q_* polymorphisms, with *PON1_192Q_* being much less efficient in CPO detoxification. Mice expressing this latter alloform have been developed and represented a valid mouse model of increased vulnerability to the effects of OP compounds [216] in adulthood, where effects of OPs were magnified in *PON1* knockout mice. Contrary to expectation, effects of the genotype were rather mild in the case of developmental (postnatal) CPO exposure, limited to startle latency and transient hyper kinesis, suggesting possible effects on catecholaminergic neurotransmission [217]. Whereas this latter effect deserves further investigations by tests designed specifically to assess dopaminergic or noradrenergic function, the relatively few neurobehavioral effects associated with CPO exposure in this study also suggest that the reported neurobehavioral and biochemical consequences of CPF exposure do not entirely depend on its conversion to CPO.

Two studies [140,218] have examined the neurobehavioral changes in metallothionein *MT1/MT2*-null mice exposed to low-levels of Hg during postnatal development. As described previously, metallothioneins are central for the metabolism and detoxification of Hg. Both studies showed that deletion of the murine *MT1* and *MT2* genes slightly enhanced the behavioral impairments caused by low-level Hg exposure.

The developmental neurotoxicity of PBDE flame retardants has been explored in *in vitro* models [219]. Since PBDEs potential developmental neurotoxicity is thought to be related to increased oxidative stress, these authors have studied the effects of DE-71, a penta-BDE mixture in primary neurons and astrocytes obtained from wild-type and *Gclm* knockout mice, which lack the modifier subunit of glutamate-cysteine ligase and thus have very low levels of glutathione (GSH). This study showed that the *in vitro* neurotoxicity of the penta-BDE mixture DE-71 in neurons and astrocytes is significantly modulated by intracellular GSH levels. This may be of relevance, as individuals with genetic predisposition leading to low GSH levels may display a higher susceptibility to PBDE effects. Several polymorphisms in glutamate–cysteine ligase have been described in humans [220,221] associated with low levels of GSH; the *Gclm* (−/−) mouse thus represents a useful animal model, suitable for further *in vitro* as well as *in vivo* studies of developmental neurotoxicity in genetically-modulated vulnerability to oxidative stress. Interestingly, mice with deletions of the glutathione-S-transferaseM1 (*GSTM1*) gene presenting lower anti-oxidant defenses are more vulnerable to the neurobehavioral effects of VPA administered in pregnancy [222].

BTBR T+tf/J (BTBR) is an inbred mouse strain that displays several behavioral traits relevant to autism, Unlike transgenic knockout mouse models, whose altered phenotype may be causally related to diminished or absent expression of single major genes, the impaired sociability of BTBR mice may reflect subtle epistatic interactions within a network of related genes, many of which may be normal polymorphisms [223]. This mouse line is endowed with outstanding reproductive/breeding performances among the mouse ASD lines and has been so far used twice in studies of prenatal exposure to environmental factors. In the first study, BTBR dams were exposed to a maternal immune activation (MIA) challenge, *i.e.*, a gestational treatment with poly (I:C) that mimics maternal viral infection known to induce autism-related behavioral alterations in the offspring [224]. In BTBR mice, MIA effects appear more robust and potentiated in some (not all) of the behavioral patterns analyzed, suggesting that combination of genetic factors and a fetal insult may exert synergistic effects leading to a more pervasive behavioral phenotype [225]. In a second study, pregnant BTBR females received sub toxic doses of CPF from gestational day 14 to 17. The results of this study highlight a specific vulnerability of BTBR mice to CPF, with delayed motor development in mouse pups exposed to the OP insecticide, an effect not previously evidenced in other mouse strains [226].

### 8.2. Stress and Environmental Contaminants in Animal Models

Many more studies have focused on the interaction and the combined action of factors such as stressful life events—occurring during perinatal period directly on fetus or mediated by mother—with chemical exposure, combining developmental exposure to neurotoxic agents with maternal stress. An exhaustive review on this topic has been published in 2007 [227]: the authors reviewed 36 studies implicating gestational stress (mostly produced by physical restraint of the pregnant female rats/mice) and chemical exposure to different classes of chemical (including metals), considering different developmental toxicity endpoints (*i.e.*, resorption, fetal viability, somatic growth of the offspring, incidence of malformation, reflex development, apoptosis in brain tissue). In general, developmental toxicity of most of the chemicals analyzed is exacerbated by association with maternal stress; however, the magnitude of the adverse effects significantly varied, depending on the dose of the chemical as well as on the severity and timing of maternal stress.

More recent studies showed that chemical exposure to Pb, MeHg and diesel exhaust can act synergistically to modulate behavioral and neural toxicity in offspring with shared underpinning biological substrates. Specifically, in rats co-occurring exposure to MeHg and stress during pregnancy resulted in neurobehavioral alteration in the offspring at adulthood. Behavioral changes were associated with alteration in levels of serum corticosterone and monoamine in different regions of the brain. Overall changes in behavior and monoamine pathways seem to depend on the sex of the offspring [228]. Other studies have considered the effects of a single dose of MeHg and maternal restraint stress showing fetal resorption and decrease of maternal weight gain only at high doses (25 mg/kg) [229,230].

The interaction between stress in pregnancy and chemical exposure has been extensively studied in the case of metals. As for arsenic exposure, teratogenic combined effects of sodium arsenate and maternal restraint during early gestation (GD 9) consisted of reduction of fetal weight in the combined arsenate and restraint group only [231]. Effects on fetal viability were evident in a subsequent study of maternal restraint and prenatal exposure to arsenic during the late gestational phase (GD 15–18), together with a delay on offspring postnatal development (*i.e.*, later pinna detachment and eye opening and earlier decline of pivoting behavior, which normally persists until postnatal day 9) [230].

A series of studies carried out on rats demonstrated the neurotoxic synergic effect of lead (Pb) exposure during pregnancy in combination with prenatal stress and suggests biological unified mechanism for their effect on health. In particular, joint exposure to Pb and maternal restraint during gestation can induce additive changes in glucocorticoid negative feedback measured in terms of serum corticosterone levels after acute stimulation with dexamethasone [232]. Other studies have considered the effects of the interaction between blood Pb levels and prenatal stress on mesocorticolimbic system of the brain [233,234,235]. A prolonged Pb exposure (from two months before gestation throughout lactation) with restraint stress on late gestation (GD 16–17) enhanced the toxic effects in the rat offspring differently in the two sexes with rather unpredictable patterns of changes in monoamine neurotransmitters, corticosteroid systems, and/or behavior. In male offspring, Pb exposure *per se* increased corticosterone levels, whereas in females only the combination of both maternal stress and Pb exposure increased corticosterone levels [233]. Remarkable results were also found in rats exposed in the early postnatal period to chronic stress (barren cage rearing) and Pb. Both stressors changed serotonin and norepinephrine levels as well as dopamine and serotonin turnover also in interaction, though no combined effect on corticosterone was found [236].

In mice, intermittent exposure via oropharyngeal aspiration to diesel exhaust combined with standard housing or nest material restriction during the last third of gestation induced long-term behavioral changes in the offspring, increasing anxiety level in males. Furthermore, diesel exposure combined with maternal stress decreased anti-inflammatory IL-10 in male and increased expression of the innate immune recognition gene toll-like receptor 4 (*Tlr4*) and its downstream effector, caspase-1 [237].

As for OP pesticides, prenatal exposure in rats to dexamethasone to mimic gestational therapeutic administration in pre-term labor enhanced behavioral alterations due to early postnatal exposure to CPF and measured at adolescence and adulthood, suggesting that prenatal glucocorticoids (likely modulated by maternal stress) can generate a subpopulation with enhanced vulnerability to environmental toxicants [238].

Overall, experimental models of co-occurring exposure of chemical and non-chemical stressors show that they can impact the same physiological target pathways, though additive or synergic interactions do not occur by default. Variables such as route and time of exposure of chemical and stress as well as the sex should be accurately taken into account in the assessment of cumulative risk. As for route of exposure, developmental toxicity studies with bisphenol-A have recently suggested that oral gavage *per se* includes a maternal stress-component able to modulate the effects of this chemical, representing a confounder of which not all researchers are aware [239].

## 9. Designing *in Vivo* Experiments to Model Complexity

One of the aims of laboratory research in behavioral toxicology should be that of identifying and validating biomarkers, to empower the exposome approach with a set of peripheral accessible markers of susceptibility, vulnerability and effect, able to predict the neuropsychological outcome.

To this aim, experiments performed in animals should consider different exposure factors, modeling human exposure scenarios. Together with the toxicant under study, factors mimicking demographic and socio-economic variables usually considered in humans should be included (e.g., sex, age, maternal stress, rearing conditions, environmental enrichment). The inclusion of these factors implies a greater complexity of the experimental design and statistical analysis, and it can produce higher variability of the outcome measures, rendering it more difficult to highlight the effect of the toxicant in study. However, it would increase generalizability of the results and thus give greater translational value to the study performed.

One important opportunity of modeling developmental neurotoxicity is the possibility to discriminate different size effects in association with different dosages. This allows to describe more than one scenario of human exposure and to detect dose-effect response: from the dosage that triggers very subtle effects to the limit dose which still allows reproduction and viability of individuals though in the presence of impairment in other domains.

Use of genetically-modified mouse lines carrying common and potentially functional polymorphisms is recommended to verify the impact of contaminants, always considering the matter of validity when a particular genetic model is chosen. Especially with the aim of testing susceptibility and in particular its gene-environment basis, the use of genetic model often makes disease phenotypes stable and well defined, which offer low resolution for the effects of environmental factors added in the experimental setting to study interaction. The use of heterozygous lines is more promising in this perspective as well as natural strains harboring specific polymorphism. They model a human genetic condition that is more frequent and more difficult to be detected (being often asymptomatic) than homozygosity, but still may result in increased vulnerability to toxicant effects with respect to wild-type strains. 

Dealing with experimental models of NDDs the “time” factor should be considered twice, both in terms of critical time windows of susceptibility and in terms of age of the subjects involved in the study. Indeed, there are many cases of critical periods of vulnerability of developing organisms (during prenatal phase, infancy, adolescence) to so called “sub toxic” doses of environmental contaminants, chlorpyrifos being a paradigmatic example in this context [240].

Age of the subject is also a critical factor, and since by definition NDDs refer to developing individuals, the neurobehavioral outcome should be assessed at different life stages, obviously including neonatal and/or juvenile period to identify early alterations potentially predictive of later behavioral deficit.

In our experience, the selection of behavioral tests to be used in such studies should include different domains (cognitive, emotional, social profiles) to increase both the total informative value of such analysis (generalized *vs.* more selective effects) and level of comparability with the neuropsychological test batteries used to assess infants/children. Again in this framework the requirement of early behavioral assessment is mandatory, to fully integrate the results of *in vivo* studies in animal models with those of epidemiological birth cohort studies on the role of neurotoxicant in NDDs.

In parallel with the characterization of the behavioral effects of the chemical in study, omic biomarkers (microRNAs, DNA methylation, and protein and metabolite levels) should be measured in peripheral organ/tissues such as the blood and in the brain at different time points, to evidence parallel changes and possible differences, eventually to validate the peripheral biomarkers as indexes of a CNS disorder. Once the effect of the contaminant in the genetic model has been characterized in multidose experiments, one may verify the effects on the behavioral outcome of adding to the chemical in study a “non-chemical” stressor such as mild stress in pregnancy or maternal inflammation.

A last methodological caveat concerns the experimental design and related statistical methods: in experiments on developmental toxicology, pups are usually exposed to toxicants through the mother either prenatally (*in utero*) or postnatally (via lactation). In both cases, neurotoxicants, that are primarily administered to mothers, can affect the offspring neurodevelopment not only directly by reaching the target systems (nervous or neuroendocrine), but also indirectly, interfering with dam’s maternal care in both qualitative and quantitative terms [241]. Early experiences of pups have been shown to influence epigenetically the nervous and endocrine development, and hence must be taken into appropriate account to better understand causes and pathways of toxicant effects on developmental outcomes [242]. Experimental design and the statistical analysis of data can address this issue. As for the experimental design, the sample size must be calculated with respect to the litters and not to the individual pups (since littermates that are reared by the same dam cannot be considered as independent statistical units, their response to stimuli being strongly influenced by the maternal environment). In addition, littermates must be balanced as well as possible for all within-litter factors under study (e.g., sex and repeated testing). With respect to the statistical analysis, the litter must be included as a statistical unit for all between-subject factors (such as prenatal treatment(s) and rearing conditions), and as random blocking factor for all within-litter factors (such as sex, and age in case of repeated testing on the same statistical units). Statistical analyses that do not take into appropriate account the litter effect can be strongly biased in whatever direction, potentially leading to either an overestimation or an underestimation of treatment effects: this has been repeatedly pointed out in the past [243,244], but these methodological warnings keep on being often neglected in current literature of NDD mouse models as recently illustrated for prenatal VPA studies [245].

## 10. Conclusions

Strong evidence exists that environmentally relevant exposure to chemical pollutants at critical developmental stages affects neural and behavioral development in children. Recent advances in research offer important clues into pathogenetic mechanisms of autism and other NDDs, indicating that environmental risk factors cannot be ruled out. Specifically, in NDDs, variations in several candidate genes may confer higher vulnerability to different kinds of adverse environmental stressors, including early exposure to chemicals. Although there is a lot of information derived by epidemiological data that support the adverse effects of many chemicals on child neurodevelopment there are still large gaps in knowledge in this field, as for the mechanisms of toxicity, the estimation of the dose-response relationship and the individual dimension of the exposure history. Furthermore, the large number of studies investigating the role of gene polymorphisms, epigenetic mechanisms, nutritional and socioeconomic factors in the determination of individual vulnerability to chemical exposure has not substantially advanced our knowledge of the complex etiology of NDDs and brain disorders in general.

All this considered, there is an overall need to invest more in discovery research able to fill out the knowledge gaps which lay beneath neurotoxicological hypothesis for NDDs. Along this line, the exposomic approach is aimed at characterizing and quantifying the exogenous and endogenous exposures and modifiable risk factors that predispose to and predict NDDs. A critical issue in such context is the identification and validation of peripheral biomarkers of effects that can inform on typical and atypical brain development, and help to establish biologically plausible links between chemical exposure and health effects. This requires a trans-disciplinary approach bridging several scientific fields such as epidemiology, environmental and exposure science and toxicology, to collect data from different biological sources and organizational levels. 

Behavioral toxicology studies might significantly contribute to this innovative research approach, by modeling in “simpler” living organisms the complexity of the human exposure scenarios. Studies with laboratory rodents allow assessment of dose-response relationships, critical periods of susceptibility, and the relative contribution of genetic, epigenetic and environmental factors. More importantly in the neuropsychiatric disease field, the use of the *in vivo* models permits the selection of omic biomarkers anchored to the behavioral phenotype, which increases their translational value. Thus, in synergy with mechanistic *in vitro* studies [246], physiologically-based toxicokinetic (PBTK) models [247] and human observational and biomonitoring data, *in vivo* models may be pivotal to identify candidate biomarkers and pinpoint susceptible groups or lifestages to be translated to large prospective studies within the exposome context.
